# Melatonin and Fertoprotective Adjuvants: Prevention against Premature Ovarian Failure during Chemotherapy

**DOI:** 10.3390/ijms18061221

**Published:** 2017-06-07

**Authors:** Hoon Jang, Kwonho Hong, Youngsok Choi

**Affiliations:** 1 Department of Biomedical Science, Cha University, 335 Pangyo, Bundang, Seongnami, Gyeonggi 13488, Korea; hoonjang@chamc.co.kr; 2 Department of Stem Cell and Regenerative Biotechnology, Konkuk University, 120 Neungdong-ro, Gwangjin-gu, Seoul 05029, Korea

**Keywords:** melatonin, chemotherapy, fertoprotective adjuvant, premature ovarian failure

## Abstract

Premature ovarian failure is one of the side effects of chemotherapy in pre-menopausal cancer patients. Preservation of fertility has become increasingly important in improving the quality of life of completely recovered cancer patients. Among the possible strategies for preserving fertility such as ovarian tissue cryopreservation, co-treatment with a pharmacological adjuvant is highly effective and poses less of a burden on the human body. Melatonin is generally produced in various tissues and acts as a universally acting antioxidant in cells. Melatonin is now more widely used in various biological processes including treating insomnia and an adjuvant during chemotherapy. In this review, we summarize the information indicating that melatonin may be useful for reducing and preventing premature ovarian failure in chemotherapy-treated female patients. We also mention that many adjuvants other than melatonin are developed and used to inhibit chemotherapy-induced infertility. This information will give us novel insights on the clinical use of melatonin and other agents as fertoprotective adjuvants for female cancer patients.

## 1. Introduction

The most significant and common side effects of chemotherapy include infertility and premature ovarian failure (POF) [1]. Therefore, prevention of POF and protection of the ovarian follicle pool have gained increasing attention to improve the quality of life of female cancer patients receiving chemotherapy. Major international guidelines recommend that physicians should discuss with their female cancer patients at risk of chemotherapy-induced POF and ovarian dysfunction, and help with the decision of fertility preservation as early as possible [2,3,4]. Among the strategies for preservation of fertility, cryopreservation of a piece of ovarian tissue or premature oocyte is commonly considered. However, these methods are limited because of several factors such as time, cost, and gonadotoxic potential attributable to storage procedures [2,4]. Protective adjuvants that can protect the dormant follicle pool and prevent follicle loss during chemotherapy would provide considerable advantages over current fertility preservation strategies, in that they would be appropriate for young patients.

Until recently, because of the lack of understanding of the mechanism underlying the side effects of an anticancer drug, there had been limited improvement in the field of ovarian fertility preservation. However, many patients experience chemotherapy-induced side effects, and the need to preserve fertility has been repeatedly highlighted by physicians. In this brief review, we summarize the mechanisms and reports demonstrating the role of melatonin as a fertoprotective adjuvant that suppresses chemotherapy-induced dormant follicle activation and preserves the follicle reserve. The data suggest that melatonin could be a potential agent in the field of fertility preservation for chemotherapy-treated female cancer patients.

## 2. Chemotherapy-Induced Ovarian Disorder

Chemotherapy remains the standard of care for cancer, and could damage various organs depending on the age and sex of patients and dose of agents [5,6,7]. Chemotherapeutic agents inhibit vital processes of cells, thereby arresting cell proliferation, and inducing abnormal activation of the dormant follicles in the ovary. Previous studies have reported that anticancer drug treatment induces varying levels of ovarian damages, resulting in repression of fertility [8,9], and presented solutions to ameliorate ovarian atrophy and prevent the loss of follicle reserve and infertility [1,10,11]. Although older women have a lower follicle pool and are more susceptible to POF than young women [6] due to chemotherapy-induced apoptosis of somatic cells in growing follicle and fibrosis of stromal blood vessel in the ovary [9,11], burn-out of dormant follicle pool is still significant [12,13].

### 2.1. Chemotherapeutic Drugs

Several groups of anti-cancer drugs classified based on their type and action are listed below. First, alkylating molecules, such as cyclophosphamide, have significant damaging effects on ovarian tissue [14,15,16], and are responsible for the highest rates of age-related ovarian failure [12]. Second, platinum-based molecules, such as cisplatin, cause amenorrhea [17,18], and induce DNA damage in the ovary through c-Abl tyrosine kinase inhibition and p63 activation [19,20]. Cisplatin also induces aneuploidy in oocytes and early embryonic death [21]. Third, anthracycline compounds such as doxorubicin induce oxidative stress in mature/premature oocytes and trigger dominant lethal mutations and aneuploidy [22]. Clinical data indicate that doxorubicin has an intermediate or a lower risk than that of other chemotherapeutic agents [12,23]. However, Hortobagyi et al. reported that the frequency of amenorrhea was much higher in women older than 30 years who received doxorubicin treatment (33% in women aged 30–39-years, and 96% in women older than 40 years) than in those younger than 30 years [24]. Fourth, vinca alkaloids such as vinblastine induce a high level of aneuploidy in an animal model [22], but a clinical study reported a reduced risk of ovarian failure [25,26]. Fifth, anti-metabolites such as methotrexate and 5-fluorouracil do not affect fertility; however, data regarding this are limited [27]. One of the anti-metabolites, methotrexate is generally used to treat ectopic pregnancy without any agent-related side effect [28,29]. Sixth, the effects of taxane family-related drugs such as paclitaxel on fertility are controversial. Several studies have suggested low or no risk of amenorrhea [30,31,32,33,34], whereas other studies reported gonadal toxicity as indicated by high follicle stimulating hormone (FSH) levels [9] and an increasing risk of amenorrhea [32,35]. Finally, biological targeted therapy is a novel form of chemotherapy and comprises drugs such as tamoxifen or herceptin that interfere with specific factors expressed by cancer cells. Because it has only recently been used to treat tumor cells and designed to target specific cancer cells, there are fewer data on its effects on the ovary. Therefore, these agents are mostly used as therapeutic adjuvants after initial chemotherapy due to their fewer side effects and low risk on the fertility [36]. However, additional clinical data and studies are necessary. Patients are often treated with the above-mentioned agents and their combinations, but it is difficult to predict ovarian damage due to inter-individual variations [12,37]. A combination of adriamycin, bleomycin, dacarbazine, and vincristine for lymphoma is reported to be less ovotoxic [38,39,40], whereas another major combination of cyclophosphamide, procarbazin, prednisone, and vincristine induces POF [41,42]. In order to understand the mechanism of POF induced by chemotherapy, it is necessary to accumulate clinical data continuously.

### 2.2. Mechanisms of Chemotherapy-Induced Ovarian Disorder

Anticancer drugs induce DNA damage or inhibit cell division, eventually leading to cell apoptosis. In the ovary, DNA damage, such as DNA double-strand breaks, induced by chemotherapy activates apoptosis of somatic granulosa cells and oocytes [43,44,45]. In addition, chemotherapy stimulates abnormal activation of dormant primordial follicles, leading to POF [1,27,46,47]. The response and reaction of chemotherapy agents depend on cells. Therefore, understanding the signal transduction and mechanisms of chemotherapy agents is important for preserving germ cells in the ovary.

The toxic effect of chemotherapy on dormant primordial follicles is not clearly studied, whereas it has been demonstrated that anticancer drugs induce apoptosis of oocytes and somatic cells such as granulosa and cumulous in large follicles [20,21,43,48]. Oktem and Oktay reported that cyclophosphamide rapidly decreases the number of primordial follicles and induces apoptosis in a human-mice ovarian xenograft model [49]. In vitro ovarian culture showed that spontaneous activation of primordial follicles is stimulated by chemotherapeutic agents in animals [50] and humans [51]. Recently, Xiang et al. reported that cyclophosphamide, an alkylating molecule, induces dormant follicle pool depletion by indiscriminately activating primordial follicles without inducing death, leading to the induction of POF [52]. Cisplatin, a platinum-based agent, also strongly triggers activation of primordial follicles without inducing follicle death [53,54].

**Figure 1:**
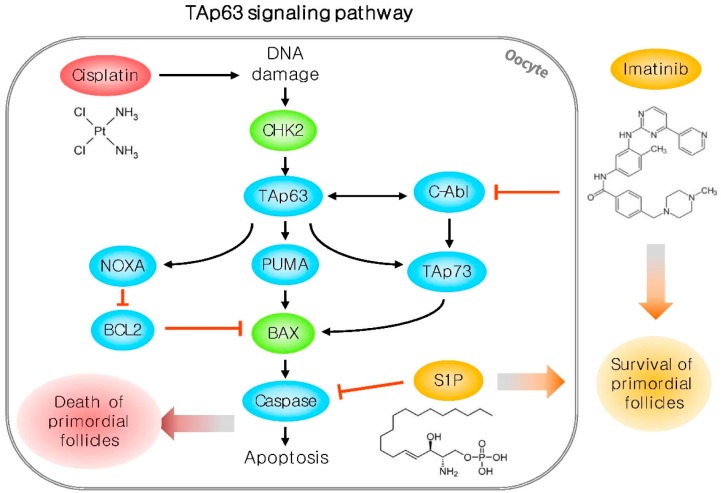
Schematic model for chemotherapy-induced oocyte death via the TAp63 signaling pathway. Cisplatin treatment causes DNA damage in oocytes and induces a surveillance factor, Chk2, followed by activation of NOXA, PUMA, and TAp73. Therefore, DNA-damaged oocytes undergo programmed cell death. However, imatinib and S1P rescue these DNA-damaged oocytes via inactivation of the TAp63 signaling pathway. Black arrow: activation; red T bar: suppression.

Two main pathways explain how the primordial follicles in the ovary die or are activated. One is the tumor suppressor protein TP53 (known as p53)-dependent pathway. TP53 has been reported to be involved in apoptosis of ovarian granulosa cells in rats [55,56]. Conversely, Depalo et al. showed that TP53 protein is less expressed in human ovarian follicles [57]. An anticancer drug, doxorubicin did not stimulate apoptosis of mature oocytes in *TP53*-deficient mice [58]. These suggest that the mechanisms of TP53-mediated follicle apoptosis and chemical-induced DNA damage are independent of each other. Recently, several studies demonstrated that TAp63, a homolog of TP53, is present in the nucleus of oocytes [59] and is a master regulator of DNA damage or repairing system in the oocyte of primordial follicles [60,61]. When TAp63 is lacking, oocytes exhibit resistance to radiation-induced DNA damage [60]. Additionally, p53-upregulated modulator of apoptosis (PUMA) and Phorbol-12-Myristate-13-Acetate-Induced Protein 1 (NOXA) play an important role in regulating downstream factors in the TAp63 signaling pathway in DNA-damaged oocytes [62]. The regulator of TAp63 transcribes c-Abl tyrosine kinase, which maintains genomic integrity by regulating DNA status in cells [63]. c-Abl and TAp63 regulate apoptosis of cells exposed to chemotherapeutic agents such as cisplatin [20] and doxorubicin [64]. Kim et al. showed that cisplatin induces c-Abl and TAp73, another homolog of TP53, in ovaries [65]. In addition, Bolcun-Filas et al. reported that checkpoint kinase 2 (Chk2) is essential for surveilling and killing oocytes undergoing abnormal meiosis and harboring DNA double-strand breaks [66]. A further study is needed to determine whether checkpoint kinase 2 (CHK2) is regulated by anticancer drugs. However, the results of the existing studies suggest that CHK2 and TAp63 regulate crucial pathways via PUMA, NOXA, bcl2-associated X protein (BAX), TAp73, and c-Abl (Figure 1).

**Figure 2:**
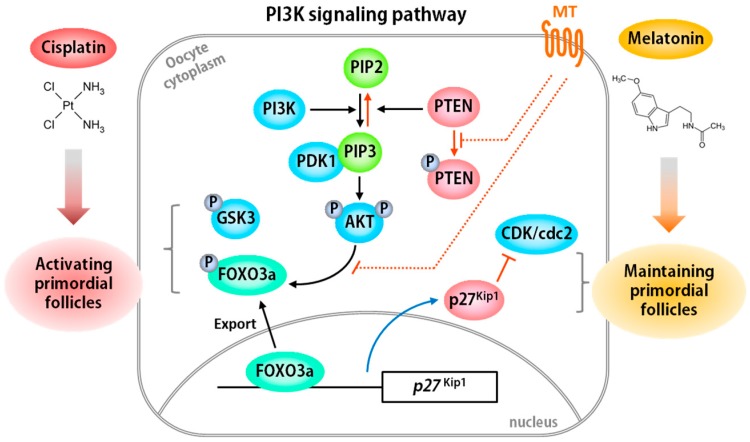
Schematic model for regulation of primordial follicle activation via the PI3K signaling pathway. Cisplatin induces activation of the PI3K/AKT/GSK/FOXO3a pathway via a phosphorylation cascade leading to activation of dormant primordial follicles. However, melatonin suppresses cisplatin-induced activation by inducing PTEN activity and inhibiting FOXO3a phosphorylation, thereby resulting in the expression of p27^Kip1^, CDK inhibitor, during chemotherapy. Black arrow: activation; red T bar: suppression; dotted line T bar: possible inhibition; red arrow: inactivation; blue arrow: transcription.

The other regulatory pathway is the PI3K-dependent signaling pathway. Various studies have shown that the activation of dormant follicles in the ovary is regulated by the PI3K/PTEN/AKT signal pathway in mice [67,68,69,70], and in a human in vitro model [71,72]. The balance of factors within the PI3K pathway is very important for determining the fates of dormant primordial follicles [73]. PTEN is a key negative regulator of the PI3K signaling pathway and plays critical roles in various tissues and cells [67,68,74,75,76,77]. AKT is activated by phosphorylation of the PI3K signaling pathway and phosphorylates downstream signal pathway proteins including forkhead box O3a (FOXO3a), glycogen synthase kinase (GSK), tuberous sclerosis 1/2 (TSC1/2), and bcl2 associated against of cell death (BAD) [53,70,73,78,79,80]. Anticancer drugs activate the PI3K/AKT signal pathway, which results in continuous activation of dormant primordial follicles causing POF during chemotherapy [81]. FOXO3a is an important transcriptional factor in the PI3K signaling pathway and regulates primordial follicle activation [82]. When a primordial follicle is activated, FOXO3a is phosphorylated and exported from the nucleus to the cytoplasm [53]. In the nucleus, FOXO3a functions as a transcriptional activator to induce the expression of *p27*^Kip1^, which encodes a cyclin dependent kinase (CDK) inhibitor protein for maintaining quiescence of primordial follicles [83,84]. In fact, the deficiency of *p27*^Kip1^ results in excessive activation of primordial follicles leading to POF in mice [85]. The molecular pathway of PI3K signaling in the regulation of primordial follicle fate is summarized in Figure 2. Therefore, it is critical to understand the molecular mechanism of the PI3K pathway rather than that of the TP53 pathway and to find appropriate fertoprotective adjuvants to protect the activation of follicle reserve in the ovary during chemotherapy.

## 3. Melatonin and Other Adjuvant Agents for Protection against Chemotherapy-Induced Follicle Depletion

### 3.1. Melatonin

#### 3.1.1. Pleiotropic Effects of Melatonin on Cancer Prevention and Immune System

Melatonin (*N*-acetyl-5-methoxytryptamine) is primarily revealed as a secretory product of the pineal grand in vertebrates, and is a derivative of tryptophan [86]. Recently, it has been shown that melatonin is produced in various tissues including reproductive tissues such as ovary and placenta [87,88,89,90,91]. This molecule is lipophilic, and acts as an antioxidant and a free radical scavenger [92,93,94,95,96,97]; it is present in many biological fluids such as synovial fluid, amniotic fluid, cerebrospinal fluid, saliva, bile, and breast milk [98,99]. Several studies have reported that exogenous melatonin has protective effects in the kidneys [100,101], nerve system [96,102], lungs [103], ovaries [46], uterus [104,105], and testes [106], and against oxidative stress [107]. Melatonin level and production gradually decrease with age [108,109], and this status can be very important for the overall decrease in the quality of life of the elderly [110]. Other reports have shown that a decreasing level of melatonin is relevant to the development of various diseases [111,112], and that supplementation of melatonin improves the quality of life of the elderly and patients [113,114]. These imply that melatonin is a pleiotropic molecule modulating cellular response spatially and temporally.

Melatonin has been focused to be an anti-cancer agent as well as antioxidant via regulating various cellular mechanisms including cell proliferation and angiogenesis. First, melatonin can prevent cancer growth. In 2000, Mocková et al. reported an interesting paper that melatonin administration suppressed chemocarcinogen-induced mammary carcinogenesis [115]. Yousefi and colleagues reviewed recent studies about melatonin effect on the regulation of DNA damage response and repair [116]. In response to DNA damage response, a protein kinase, ataxia-telangiectasia mutated (ATM) activates DNA damage checkpoint resulting in apoptosis and senescence. Melatonin reduced radiation-induced DNA damage by suppressing ATM expression [117]. In addition, there are several key regulators such as p53 and p21 induced by DNA damage response which are involved in cell cycle arrest. Several studies showed that melatonin suppressed proliferation of cancer cells such as breast cancer cell line, MCF-7 (Michigan Cancer Foundation-7) and hepatocarcinoma cell line, HepG2 by activation of p53 and p21 pathway [118,119,120,121]. Phosphorylation of p53 at serine 15 residue (Ser-15) is important for the p53 activity against DNA damage. Santoro and colleagues demonstrated that melatonin induces p53 phosphorylation at Ser-15 residue and that melatonin preventing effect on DNA damage is mediated via melatonin receptor (MT) [122,123]. There are two types of melatonin receptor, MT1 and MT2 in mammals [124]. Melatonin receptors are G protein-coupled receptors, which play an important role in various cellular processes and drug responses [125]. Santoro et al. showed that melatonin activates p38 MAPK-dependent phosphorylation of p53 [122] and the signaling pathway is mediated by melatonin receptor [123]. Indeed, the phosphorylation of p53 is independent of ATM.

Secondly, melatonin is able to inhibit tumor growth by suppression of angiogenesis. Several reports demonstrated that the angiogenesis is prevented via decreasing the expression of endothelin converting enzyme-1 [126] and reducing the activation of vascular endothelial growth factor (VEGF) receptor 2 (VEGFR2) [127,128]. Recently, the anti-angiogenic effect of melatonin was proved in xenograft models of breast cancer [129]. They demonstrated that melatonin effectively reduced tumor growth and cell proliferation by the inhibition of angiogenesis. An interesting study recently reported that melatonin can seasonally control pituitary function by regulating the expression of VEGF and VEGFR2 in the pituitary. They showed that melatonin controls producing isoform type of VEGF and VEGFR2 resulting in angiogenesis modulation which is critical for hormone release of the pituitary depending on breeding season [130].

Thirdly, the immunomodulatory effect of melatonin in cancer was reviewed by Miller et al. in 2006 [131]. Melatonin can activate T-lymphocyte [132,133,134] macrophage [133], as well as cells of spleen [135], lymph node and bone marrow [136]. In addition, melatonin stimulates the production of cytokines including interleukin (IL)-2, interferon (IFN)-γ and IL-6 [137,138]. Another effect of melatonin on immune system is to regulate tumor immunosurveillancers such as natural killer (NK) cells [139]. Melatonin treatment in leukemia model increased the number of NK-cells [140]. A recent report showed that melatonin can improve the activity of T-lymphocyte which was decreased in aged mice [141]. These imply that melatonin is able to enhance the immune system via production of cytokines leading to prevention of various cancers.

#### 3.1.2. Melatonin as Antioxidant in Ovarian Follicles

Melatonin acts as a powerful antioxidant to prevent free radical damage by oxidative stress in the body. The oxidative stress causes various diseases including cancer, neurological disease, rheumatoid and reproduction [142,143,144]. The early ovarian follicle loss causes premature ovarian failure in premenopausal women. The primordial follicles during reproductive life have three fates: dormancy, activation, and atresia [145,146]. Most primordial follicles should be quiescent until activation for oocyte maturation. Once recruited and activated, the maturation of oocytes proceeds very rapidly. This dynamic process causes considerable oxidative stress. Tamura et al. reported that the oocyte quality was lowered by oxidative stress [147]. In fact, the follicular fluids contains significant amount of melatonin [148]. Several studies have reported that melatonin in ovarian fluid protects the oocytes and granulosa cells by ameliorating oxidative stress during ovulation which is critical for normal maturation [147,148,149]. In addition, the supplement of melatonin improves oocyte quality [147,150,151]. Interestingly, melatonin receptors (MT1 and MT2) are expressed in the ovary [152,153,154]. These suggested that melatonin acts as a scavenger via its receptor. Recent study demonstrated that melatonin treatment ameliorates premature ovarian failure by decreasing oxidative stress damage, which was mediated by SIRT1 signaling [151]. Of course, the effect of melatonin on the primordial follicles remains unclear. However, these imply that the ovarian melatonin might act regionally in the primordial follicles from dynamic oxidative stress during folliculogenesis.

#### 3.1.3. New Application of Melatonin as a Fertoprotective Agent in Fertility Preservation

Many anti-cancer drugs including cyclophosphamide and cisplatin have been known to induce apoptosis of granulosa cells in the ovary and stimulate over-activation of dormant primordial follicles resulting in premature ovarian failure (POF) [1,27,43,44,45,46,47]. POF is one of the causes of female infertility. Fertility preservation of cancer female patients is very important for their life after cancer survival. Therefore, it has become important for finding a fertoprotective agent which protect germ cells and increase the efficacy of anti-cancer drug during chemotherapy. Interestingly, melatonin has various prospects as a potential therapeutic adjuvant during chemotherapy. The treatment of melatonin reduces the adverse effects of chemotherapy by removing superoxide anion, hydrogen peroxide, and peroxyl radical [155,156,157,158,159]. Several studies have demonstrated that melatonin treatment protects depletion of germ cells in the gonads during chemotherapy. In male reproductive organ, melatonin administration prevents cisplatin-induced testicular toxicity and reduces sperm motility [106]. Chang et al. showed that cisplatin induces the depletion of follicles via over-activating the dormant primordial follicles in the ovary [54]. They examined the protection effect of melatonin on cisplatin-treated ovaries. Combined treatment with melatonin and cisplatin significantly prevented primordial follicle loss in cisplatin-treated ovary. Recent reports give us a clue for the molecular mechanism of melatonin in the ovary. As mentioned above, melatonin signals through two types of melatonin receptors, MT1 and MT2 in mammals [124]. In fact, several studies supported that melatonin receptors are present in the oocytes and granulosa cells of various species ovary including human [154,160,161,162,163]. Melatonin receptors are G protein-coupled receptors, which play an important role in various cellular processes and drug responses [125]. Therefore, the protective effect of melatonin in follicles is thought to be achieved through the G protein-coupled receptor-dependent pathway (Figure 2). In 2016, Jang et al. demonstrated that the regulatory protection effect of melatonin is mediated by suppressing the activation of the PI3K/AKT/FOXO3a signaling pathway in cisplatin-treated ovary [53]. This suggests that melatonin is directly involved in PI3K signaling via its receptor. The theory is supported by two reports. One is showing that melatonin induces AKT phosphorylation PI3K signaling pathway in astrocyte [164]. The other explained that melatonin mediates neuroprotective activity in ischemia via PI3K/PTEN/AKT signaling pathway [165]. In addition, the ovary can produce melatonin [148]. Follicular fluid in the growing follicles contains high level of melatonin [166,167]. The supplementation of melatonin for in vitro maturation of oocytes improves the oocyte quality [150,167]. These indicate that the endogenous melatonin is not enough for preventing chemo-induced primordial follicle loss in the ovary even though it is critical for oocyte development. The detail molecular mechanism of melatonin protective response against chemo-induced ovarian damage needs further studies.

### 3.2. Other Candidates as a Fertoprotective Agent

#### 3.2.1. Sphingosin-1-phosphate

Sphingosin-1-phosphate (S1P) is derived from sphingolipids. Morita et al. firstly reported that S1P has a protective effect against oocyte apoptosis in radiation-induced ovary [168]. It is an anti-apoptotic agent that inhibits apoptosis through the sphingomyelin pathway, which was reported to be responsible for the death of ovarian follicles (Figure 1) [58,168]. However, the effect of S1P on the ovary is controversial. Treatment with S1P showed a protective effect against dacarbazin- [169], cyclophosphamide- and doxorubicin-treated ovarian follicles [170], but not against cyclophosphamide-treated ovary [171]. In addition, the administration of S1P during chemotherapy has some limitations. S1P treatment interferes with the clinical effects of anticancer drugs, and its anti-apoptotic effect may suppress the normal atresia of DNA-damaged oocytes during folliculogenesis. Recent studies reported that melatonin suppresses liver damage by rabbit hemorrhagic disease virus [172], and diethyinitrosamine-induced hepatic carcinoma in mice [173] by inhibiting sphingosine kinase/S1P signaling pathway. In addition, the suppressing effect of melatonin on chemical-induced S1P signaling pathway was discovered in human hepatic cells [174]. However, the effect of melatonin and the S1P signaling pathway in the ovarian follicle activation was not studied yet. Therefore, further studies are needed to discover the efficacy of melatonin in S1P signaling mechanism.

#### 3.2.2. Imatinib

Imatinib is a tyrosine-kinase inhibitor and has been proposed as a fertoprotective adjuvant to prevent dormant follicle loss induced by cisplatin treatment via inhibition of c-Abl kinase [20,65]. Kim et al. demonstrated that cisplatin induces TAp63-dependent expression of c-Abl and TAp73 resulting in activation of BAX expression in the ovary [65]. The activation of BAX is mediated by c-Abl/TAp73/BAX, leading to the death of cisplatin-damaged oocytes during chemotherapy [65]. However, there are several controversial reports that imatinib could not protect dormant oocytes from cisplatin-mediated apoptosis and prevent loss of fertility [175,176]. Because of contradictory studies on imatinib, a further study is required on the protective effect of imatinib against DNA damage in oocytes during chemotherapy. Until now, no studies have been reported on the relationship of melatonin and imatinib in certain tissues and cells. If the precise mechanism of imatinib for fertoprotective effect of ovarian follicle is established correctly, future studies on the association with melatonin will be important as a key to the resolution of ovarian follicle protection conundrum by anti-cancer drugs.

#### 3.2.3. Tamoxifen

Tamoxifen, an antagonist of estrogen receptor, is used as an adjuvant in hormone-sensitive chemotherapy. The administration of tamoxifen significantly decreased chemotherapy-induced follicle loss as well as improved fertility [177]. In addition, it ameliorated doxorubicin-induced DNA fragmentation in mouse oocytes [177], although the detailed mechanisms of tamoxifen-mediated protection during chemotherapy have not been discovered. In the studies linked to melatonin, tamoxifen had been reported as inducing tumor regression with co-treatment of melatonin in metastatic breast cancer patients [178,179,180]. In addition, melatonin enhanced the ability of tamoxifen to prevent free radical-induced damages in rat hepatic microsomes [181]. However, Dauchy et al. reported that light-induced melatonin secretion induced intrinsic resistance to tamoxifen therapy [182]. These suggest that further studies are needed to demonstrate the relationship between melatonin and tamoxifen in reproductive organs including the ovary.

#### 3.2.4. GnRH Analog

Several studies demonstrated that the administration of a GnRH analog decreased primordial follicle loss after chemotherapy in an animal model [15,183,184,185]. A clinical study showed that 281 breast cancer patients who received chemotherapy with a GnRH analog showed significantly attenuated POF [186]. However, two trials with breast cancer patients had conflicting results. One study reported that 69% of GnRH co-administrated patients who received chemotherapy have a normal reproductive cycle [187], and another study reported no difference between GnRH analog administration group and only chemotherapy group [188]. Additional studies reported no significant change in POF incidence after co-administration with a GnRH analog [189,190,191]. Thus, not only different chemotherapy protocols but also adopting different outcome definitions could explain the difference in results [37]. Studies on the correlation of melatonin with GnRH and melatonin have been reported. Diaz et al. reported that melatonin restored basal pituitary hormone levels and responsiveness to GnRH in acyclic rat model and male testis [192,193]. However, there is no research on the relationship between melatonin and GnRH analog in gonads such as ovary.

#### 3.2.5. Ammonium trichloro (dioxoethylene-*O*,*O*′) tellurate (AS101)

A tellurium compound AS101 was originally developed as an immunomodulatory agent because it stimulates cytokines [194]. However, recent reports demonstrated that AS101 decreases toxicity in several tissues including neurons [195] and testes [196,197]. In particular, AS101 has been reported to protect testes against cyclophosphamide-induced damages and fragmentation of sperm DNA without interfering with chemotherapy effect [196,197]. In female, AS101 also has been shown to suppress follicle loss in cyclophosphamide-activated primordial follicles by inhibiting the PI3K/PTEN/AKT signal pathway during chemotherapy [198,199]. AS101 significantly prevented the burn-out of dormant follicles in chemotherapy-induced mice, and successfully preserved fertility. However, further studies are needed on the side effects of AS101 in clinics and its effect on other fertoprotective agents such as melatonin.

## 4. Conclusions

Chemotherapy-induced ovarian failure is a highly burdensome gynecological syndrome. It lowers female fertility, induces premature menopause, and causes a variety of hormonal changes in the body. The development of anticancer drugs has greatly increased the survival rate of cancer patients; however, it is important to consider the quality of life as well. Researchers have studied adjuvants that can inhibit or reduce the side effects of various anticancer drugs. In this review, we summarized the chemotherapeutic drugs and adjuvants used for preserving female fertility during chemotherapy. Recently, the efficacy of melatonin as a fertoprotective adjuvant has been reported. Melatonin is a hormone synthesized in the body and it has obvious advantages over other candidates for preserving fertility during chemotherapy. It is relatively higher safer and has less toxicity in various diseases. Consequently, melatonin may be useful in preventing or ameliorating chemotherapy-induced ovarian disorders. This information will provide better understanding for application of melatonin in oncology clinics as a fertoprotective adjuvant for female cancer patients.
